# COVID-19 Vaccine Hesitancy in the LGBTQ+ Population: A Systematic Review

**DOI:** 10.3390/idr13040079

**Published:** 2021-10-07

**Authors:** Ishan Garg, Hamza Hanif, Nismat Javed, Ramsha Abbas, Samir Mirza, Muhammad Ali Javaid, Suman Pal, Rahul Shekhar, Abu Baker Sheikh

**Affiliations:** 1 Department of Internal Medicine, Maimonides Medical Center, Brooklyn, New York, NY 11219, USA; ishangargmd@gmail.com; 2 Department of General Surgery, University of New Mexico Health Sciences Center, Albuquerque, NM 87106, USA; hhanif@salud.unm.edu; 3 Department of Medicine, Shifa International Hospital, Islamabad 44000, Pakistan; nismatjaved@gmail.com; 4 Shifa College of Medicine, Shifa Tameer-e-Millat University, Islamabad 44000, Pakistan; ramshaabbas97@gmail.com; 5 Department of Internal Medicine, Dow Medical College, Karachi 74200, Pakistan; samir.mirza1910@gmail.com (S.M.); muhammad.ali.javaid1995@gmail.com (M.A.J.); 6 Department of Internal Medicine, University of New Mexico Health Sciences Center, Albuquerque, NM 87106, USA; spal@salud.unm.edu (S.P.); rahul547843@gmail.com (R.S.)

**Keywords:** vaccine hesitancy, LGBTQ+, COVID-19, lesbian, gay, bisexual, transgender, queer

## Abstract

The coronavirus 2019 (COVID-19) pandemic has disproportionately impacted lesbian, gay, bisexual, transgender, queer (LGBTQ+) people. Despite developing safe and effective COVID-19 vaccines, LGBTQ+ communities still faces challenges due to inequitable access and vaccine hesitancy. Vaccine hesitancy is a delay in the acceptance or refusal of vaccines despite the availability of vaccination services. Various studies have explored and tried to address factors influencing vaccine hesitancy. However, the LGBTQ+ population remains under- and misrepresented in many of these studies. According to the few studies that have focused on the LGBTQ+ population, several factors influencing vaccine hesitancy have been identified, with the most common factors in studies being concern about vaccine safety, vaccine efficacy, and history of bad experiences with healthcare providers. In order to rebuild the confidence of LGBTQ+ people in vaccines, governments, healthcare policymakers, and healthcare providers need to start by acknowledging, and then resolving, these disparities; building trust; dismantling systemic suppression and discrimination; and prioritizing the inclusion of LGBTQ+ people in research studies and public health policies.

## 1. Introduction

In December 2019, a cluster of pneumonia cases was reported in Wuhan, China, by the Wuhan Municipal Health Commission [1]. In February 2020, the World Health Organization (WHO) termed it coronavirus disease 2019 (COVID-19) [2]. COVID-19 is caused by the severe acute respiratory syndrome coronavirus-2 (SARS-CoV-2) virus [3]; the spread of COVID-19 exploded globally, and on 11 March 2020, the WHO declared the COVID-19 outbreak a global pandemic [4]. As of August 2021, there had been over 216 million confirmed cases of COVID-19, including over 4.4 million deaths globally [5]. A recent online post about historical disasters, using the excess mortality model, ranked the COVID-19 pandemic among the ten deadliest pandemics in human history [6].

The COVID-19 pandemic has ravaged human life and has put a tremendous strain on economies and healthcare systems. The sexual and gender minority, including lesbian, gay, bisexual, transgender, queer (LGBTQ+ (the “+” signify all of the gender identities and sexual orientations that are not explicitly covered by the other five initials)) community, however, has borne the disproportioned brunt of this pandemic [7]. LGBTQ+ communities have been subjected to systematic and structural discrimination with prominent examples from recent history, including the HIV/AIDS epidemic response, with a similar disregard during this COVID-19 pandemic [8].

COVID-19 vaccines are our most promising option to curb the COVID-19 pandemic in the global and LGBTQ+ populations. Despite the success of developing safe and effective COVID-19 vaccines, we are not yet anywhere close to controlling the COVID-19 pandemic. Various factors, including vaccine hesitancy, vaccine production, and global vaccine inequity, remain formidable challenges that should be addressed before international efforts can have an effect on controlling the COVID-19 pandemic.

For effective healthcare policy and deployment of resources, detailed and accurate information on vaccine acceptance and vaccine hesitancy is needed in LGBTQ+ communities. However, due to systemic discrimination and exclusion of the LGBTQ+ population from socioeconomic and healthcare policies, there is a lack of robust data to guide interventions and policy changes. Here, we review the current literature on the impact of the COVID-19 pandemic on the LGBTQ+ population, reasons for, and recommendations to address vaccine hesitancy.

## 2. Methods

We screened PubMed, Web of Science, and CINAHL databases for articles describing vaccine hesitancy in adults. The inclusion criteria were: (1) survey studies among LGBTQ+, (2) studies that aimed to evaluate COVID-19 vaccine acceptance/hesitancy, (3) publication language was English. The exclusion criteria were: (1) unpublished manuscripts (preprints), (2) publication language was not English, (3) articles that were withdrawn or focused on children.

After the initial search, duplicates were removed and all included search studies were imported into EndNote online software. Two independent reviewers screened the remaining studies for inclusion based on inclusion criteria; the researchers were blinded to each other’s decisions. Rayyan software and Mendeley desktop were used. The screening was done via reading the abstracts and, if needed, by reading full-text articles. Studies published in the English language or with English translation available were included in the initial review. Once the initial screening was completed, two independent reviewers reviewed the full-text articles for final inclusion. The reviewers were blinded to each other’s decisions, and a third reviewer resolved any disputes.

Data were extracted from study documents, including study design and methodology, participants’ demographics and baseline characteristics, study country, publication journal, clinical presentation, symptoms, laboratory data, imaging data, intervention, treatment, clinical outcomes, morbidity, and mortality.

**Figure 1:**
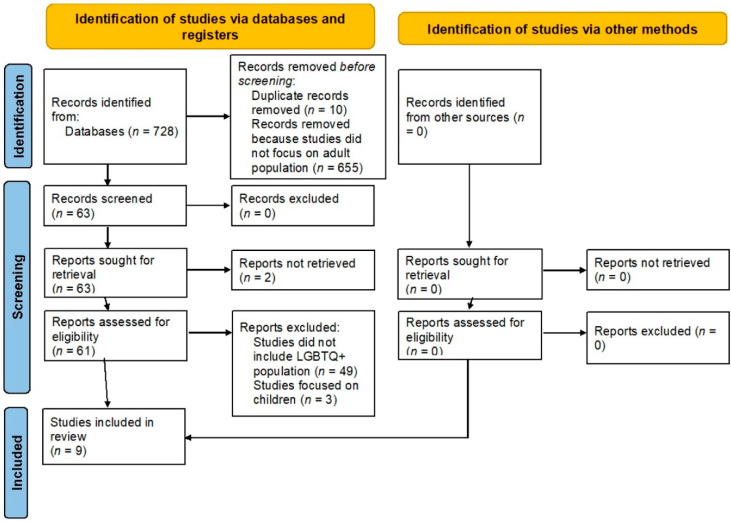
PRISMA diagram.

One reviewer extracted the data and the other reviewer cross-checked the extracted data for accuracy and completeness. The third reviewer resolved any disagreements between the first two reviewers. Attempts were made to obtain any missing data from a study by corresponding with investigators via email. If data could not be obtained, studies was excluded from the analysis on a case-by-case basis. Publications that were not peer-reviewed were excluded from this study. The Preferred Reporting Items for Systematic Reviews and Meta-Analyses (PRISMA) criteria were applied (Figure 1). The preliminary data were entered and recorded in an excel spreadsheet.

The search was completed as of July 2021, using the following strategy: (“COVID” “vaccine” “hesitancy” [Title/Abstract]) OR (“COVID” vaccine acceptance [Title/Abstract])) OR (“COVID“ “vaccine” “hesitancy” [Title/Abstract])) OR (“COVID” “intention to vaccine” [Title/Abstract]) OR (“COVID” “vaccine” “accept” [Title/Abstract]) AND (2020:2020[pdat]) OR (“COVID-19 Vaccines” [Mesh]) AND “Sexual and Gender Minorities” [Mesh]).

Articles were screened to extract data for the following: population size, option for nonbinary gender, gender, overall acceptance rate, and acceptance rate specific to the nonbinary population.

## 3. Results

The results from the review are summarized in Table 1 [9,10,11,12,13,14,15,16,17].

There were only 28 surveys (nine studies) that had a nonbinary option in the questionnaire. Among those 28 surveys (nine studies), the acceptance rate in the nonbinary population subgroup was only mentioned in three surveys (three studies) [11,13,16]. Multiple limitations made drawing any inference from those datasets challenging, including (a) inconsistent terminology to describe LGBTQ+, i.e., nonbinary, trans, other, unknown; (b) combining a nonbinary option with no response in the analysis [16]; (c) no subgroup analysis of a nonbinary group or comparison with non-LGBTQ+ population.

However, some gender-specific studies have tried to address these research-based disparities. For example, in an online surveillance-based study, Lin et al. collected data from 171 sexual minority and 876 heterosexual individuals on their intentions to receive a COVID-19 vaccination. They found that sexual minority individuals had a higher explicit and intrinsic intention to receive vaccination than heterosexual individuals [17]. Another online-based study by Teixeira da Silva et al. on COVID-19 vaccine acceptance among sexual and gender minority men and transgender women [14] collected data from 1350 individuals (predominately gay (61.6%), Black (57.9%), cisgender (95.7%) males). They found medical mistrust and social concern regarding COVID-19 vaccine stigma significantly associated with decreased COVID-19 vaccine acceptance and altruism significantly associated with increased vaccine acceptance.

In addition, LGBTQ+ supporting foundations have conducted independent studies on the impact of the COVID-19 pandemic in the LGBTQ+ population. A study based on data from an online survey by Tegan and Sara Foundation found that out of 7744 respondents, 90% identified themselves as LGBTQ+ people. They reported that nine out of ten LGBTQ+ people wanted to be vaccinated [18]. They noted that the most common concerns about getting the COVID-19 vaccine were potential side effects (59%), long-term safety (54%), and previous negative experiences with healthcare providers (19%). Previous negative experiences were more likely to delay vaccination for one in four transgender and one in three genderqueer respondents. In an online community survey among LGBTQ+ Pennsylvanians (*n* = 1534), 54.0% of the respondents reported receiving at least one vaccine dose. Of those who had not been vaccinated, 85.3% of the respondents reported a desire to be vaccinated as soon as it is available to them. Worryingly, 40.8% of the respondents did not know where to be vaccinated. They noted that the most common concerns about getting the COVID-19 vaccine were safety (59%) and effectiveness (36%) [19]. They also noted significant disparities among different LGBTQ+ groups. For instance, 57.5% Black LGBTQ+ Pennsylvanians had not been vaccinated as compared with 45.8% of all LGBTQ+ Pennsylvanians. HIV+ respondents were more likely to have received one or more vaccine doses (68.5%) than all LGBTQ+ Pennsylvanians (54.0%). HIV+ LGBTQ+ Pennsylvanians also reported a solid willingness to receive the vaccine if they had not already (82.1%).

## 4. Discussion

### 4.1. COVID-19 in the LGBTQ+ Population

LGBTQ+ communities are marginalized and systemically discriminated against, leading to socioeconomic and healthcare disparities as compared with the general population. These disparities became worse during the COVID-19 pandemic due to a combination of flagrant disregard of the LGBTQ+ community by government and healthcare policymakers and pre-existing social, economic, and health issues affecting the LGBTQ+ communities [7,8]. LGBTQ+ people may have increased exposure, economic disparities, and barriers to care as compared with cisgender heterosexual people. The Human Rights Campaign Foundation have reported that LGBTQ+ people are more likely to work in highly affected industries, often with more exposure, such as food service, hospitals, K–12 education, colleges and universities, and retail [20]. The report also noted that one in ten LGBTQ+ people were unemployed and were more likely to live in poverty than straight and cisgender people, affecting their access to affordable healthcare, including preventive measures such as testing and vaccinations. The report also suggested that the higher rates of unemployment and poverty in the LGBTQ+ population may be linked to discrimination [20]. They also noted that 17% of LGBTQ+ adults did not have health insurance coverage as compared with 12% of non-LGBTQ+ adults. Similar findings were observed by an independent nonprofit advocacy group, the Movement Advancement Project (MAP), from a poll conducted in July–August 2020 [21]. They reported that LGBTQ+ families had less secure access to financial, medical, and educational resources than non-LGBTQ+ people. It creates a self-sustaining loop of structural discrimination and subjugation. Lack of employment and education opportunities propagate poverty, homelessness, and lack of healthcare, affecting future employment and education opportunities.

In a survey-based cross-sectional study, conducted between March and June 2020, on 1380 U.S. adults (sexual and gender minority (*n* = 290) and cisgender heterosexual (*n* = 1090)), it was found that sexual and gender minority subjects had more frequent COVID-19-associated physical symptoms, as well as mental health conditions (including depression and anxiety) [22]. LGBTQ + people were more likely to have underlying comorbidities such as HIV, cardiovascular disease, and cancer than non-LGBTQ people [23,24], which may put them at an increased risk for the development of severe illness from COVID-19.

A combination of increased SARS-CoV-2 exposure risk and lack of access to healthcare has resulted in higher morbidity and mortality due to COVID-19 disease in the LGBTQ population than in the general population [7,25]. The LGBTQ+ population lost access to vital healthcare services during COVID-19, such as testing for sexually transmitted infections (STI), including HIV, and HIV preventive care including antiretroviral drugs, HIV pre-exposure prophylaxis, and access to condoms [26,27,28].

The LGBTQ+ population must not be categorized as a single uniform collective, but as communities that are as diverse as the global community itself, representing a richness of diversity in age, ethnicity, race, education, socioeconomic status, gender, sexual orientation, profession, religion, and philosophy. For instance, LGBTQ+ youth face unique challenges such as risk of physical and psychological abuse, discrimination, homelessness (with lack of safe housing and shelters due to discrimination), incarceration, poverty, and lack of education and employment opportunities (in a self-sustaining cycle) [29]. In a study, 40% of homeless youth were LGBTQ+ people [30,31].

### 4.2. COVID-19 Vaccines and Vaccine Hesitancy

Vaccines are considered to be the most promising means for preventing the spread of the SARS-CoV-2 infection and curbing the COVID-19 pandemic. This need led to the development and testing of various vaccines against SARS-CoV-2, with the approval and rollout of different safe and effective vaccines in the first half of 2021 (Moradian, 2020, “The urgent need for integrated science to fight COVID-19 pandemic and beyond” and Moradian, 2021, “Interdisciplinary Approaches to COVID-19”). As of August 2021, the WHO approved the following vaccines under the emergency use listing (EUL): (a) messenger RNA (mRNA) BNT162b2 Pfizer-BioNTech and mRNA-1273 Moderna vaccines; (b) viral vector vaccines (AstraZeneca, Janssen Ad26.COV2.S; and (c) inactivated virus vaccines Sinopharm and Sinovac [32,33]. In addition, the United States Food and Drug Administration (FDA) granted emergency use authorization (EUA) for the mRNA-based Pfizer-BioNTech and Moderna vaccines, and the viral vector-based Janssen vaccine [34]. In August 2021, the FDA gave full approval to the Pfizer-BioNTech COVID-19 (brand name, Comirnaty) vaccine to prevent COVID-19 disease in individuals 16 years of age and older [35].

Although vaccines against SARS-CoV-2 are being developed at an unprecedented pace as compared with the typical timeline for vaccine development that can range in year, the same vigorous review process has been implemented by the FDA and the WHO to maintain high safety and efficacy standards for the target human population.

To control the COVID-19 pandemic and to achieve herd immunity, from 75 to 90% of the world population needs to be vaccinated (based on various factors such as basic reproduction number, vaccine, induced immunity duration, and viral transmission in a vaccinated individual, assuming vaccine efficacy of 80%) [36,37,38]. As of August 2021, 39.5% of the world population had received at least one dose of a COVID-19 vaccine. These numbers, however, do not reflect the global inequity in vaccine distribution and availability. For instance, in the United States, over 50% of the population is fully vaccinated; however, only 1.7% of the people in low-income countries have received at least one dose of a COVID-19 vaccine [39].

To ensure the global effort to control the COVID-19 pandemic is not upended in the last mile, an optimal and equitable distribution of COVID-19 vaccines is needed. These issues need to be addressed immediately with the same zeal and ingenuity devoted to developing the COVID-19 vaccine. In addition, it is equally important to address any challenges with vaccine acceptance. Vaccine hesitancy is defined by the WHO’s Strategic Advisory Group of Experts (SAGE) Working Group (WG) as delay in acceptance or refusal of vaccines despite the availability of vaccination services [40,41].

Vaccine hesitancy is not a new phenomenon limited to COVID-19 vaccines; studies from Africa have noted a worrying trend in vaccine hesitancy in recent years [10,42,43,44,45]. Various experts have pointed out that vaccine hesitancy may become a significant factor in deciding the fate of the COVID-19 pandemic after the current bottleneck of vaccine production and distribution is addressed [46,47] (Allen, 2021, “Why are some people reluctant to be vaccinated for COVID-19? A cross-sectional survey among U.S. Adults in May–June 2020” and Arvanitis, 2021, “Factors associated with COVID-19 vaccine trust and hesitancy among adults with chronic conditions”). Although various studies and surveys from China, Southeast Asia, Japan, Ireland, Jordan, Kuwait, the USA, and the UK, are exploring the COVID-19 vaccine acceptance globally, very few studies have included or have been conducted in gender and sexual minority populations [10,11,48,49,50,51,52,53,54,55,56,57,58,59,60,61].

## 5. Factors Influencing Vaccine Hesitancy

Vaccine hesitancy is a complex and dynamic decision-making process involving various cognitive, vaccine, disease, communication, media, psychological, sociodemographic, and cultural factors [51,62,63,64]. On the basis of the epidemiologic triad of the environment, agent, and host factors, the motive behind vaccine hesitancy can be grouped into three main categories: (a) environmental (health policies, social factors, and mass media); (b) agent (vaccine safety and effectiveness, and perceived disease susceptibility); and (c) host factors (education, prior experiences, and sociodemographic and cultural factor influences) [65,66,67,68,69,70,71]. The WHO’s SAGE group identified complacency (perception of low disease risk), convenience (availability, affordability, and delivery of vaccines in a comfortable context), and confidence (trust in vaccine safety, efficacy, and competence of the healthcare system) (the 3-Cs) as the three main factors that affect attitudes towards vaccine hesitancy [40,41]. Various studies have suggested that factors including age (older individuals), education (higher education), trust in government (higher), perceived risk of COVID-19 to self (higher) and community (higher), health insurance (presence), and healthcare professionals are associated with lower vaccine hesitancy (higher vaccine acceptance) [11,49,52,55,56,57,58,72].

### 5.1. Environmental Factors (Health Policies, Social Factors, and Mass Media)

A previous study reported that healthcare workers who were hesitant to be vaccinated had poor trust in regulatory authorities and government. However, their trust in medical professionals prescribing the vaccine was somewhat higher [13]. An individual’s level of confidence in healthcare providers, healthcare policies, and healthcare services involves their perception of information provided by mass media or health authorities, as well as their perception of the adequacy of measures implemented by the government.

### 5.2. Agent-Related Factors (Vaccine and Disease)

These factors include an individual’s perception of vaccine safety, efficacy, perceived severity of disease, susceptibility to infection, and perception of one’s health status. For example, issues such as speed of development (developed and approved at an unprecedented pace) and relatively newer vaccine technology (mRNA based) may influence an individual’s perception of vaccine safety and efficacy [73,74]. Additionally, other factors, including younger age, race, and lack of trust in government, can indirectly reduce vaccine acceptance [75]. The emergence of new SARS-CoV-2 variants and COVID’s global morbidity and mortality indexes may affect an individual’s perception of disease severity and susceptibility [72,76].

### 5.3. Host-Related Factors (Personal Views)

An individual’s education, prior experiences with vaccines and healthcare, as well as sociodemographic, cultural, religious, and philosophical views (conscience objection) [77] can influence one’s decisions on vaccination. For example, the orthodox protestant groups in the Netherlands and the Amish people in the USA reject vaccination due to their religious beliefs [78].

A systemic exploration and identification of factors influencing a population and subsets of the population (such as sexual minority, transgender, African Americans, Latinos, and Native Americans) are needed to address individual concerns regarding vaccine acceptance and to help establish a framework for future healthcare policies.

## 6. Vaccine Hesitancy Studies in the LGBTQ+ Population

Considerable progress has been made in exploring various determinants of COVID-19 vaccine hesitancy, mainly using survey-based study models. However, it is crucial to note that almost all such studies have either failed to consider the LGBTQ+ population in the survey (with survey questions limited to binary gender choices and no questions on sexual orientation) or have collected insufficient information in this population subset (with no breakdown of vaccine hesitancy/acceptance rate and relationship with any factors).

## 7. Strategies to Address Vaccine Hesitancy in the LGBTQ+ Population

The systemic oppression of the LGBTQ+ population, including exclusion from research, healthcare access, and healthcare policy has led to mistrust and lack of or misinformation, contributing to vaccine hesitancy. Developing and enacting strategies addressing vaccine hesitancy is of utmost moral and clinical significance.

### 7.1. Access to COVID-19 Vaccination

Optimum production of COVID-19 vaccine doses is inadequate without it being affordable and accessible to the target population. Traditional healthcare systems and healthcare providers may seem to be inaccessible to an individual either because of one’s fear of discrimination, negative past experiences, or socioeconomic concern (concern about cost due to lack of healthcare insurance). These concerns are critical in the LGBTQ+ population. Survey data collected by the Tegan and Sara Foundation found that many queer people were hesitant or fearful of engaging with the healthcare system because of negative past experiences [18]. It should be addressed by building trust, creating safer and respectful experiences within the traditional healthcare systems, and making COVID-19 vaccines and routine SARS-CoV-2 testing available at community-based sites, including living facilities and workplaces. Having vaccines accessible in the workplace can simply help the decision-making process for an individual [79]. This approach is supported by findings observed by an LGBTQ+ health organization in Boulder, Colorado, which successfully addressed the higher rate of vaccine hesitancy among the local LGBTQ+ population by launching its own vaccine clinic, enabling people to get vaccinated in a safe space.

### 7.2. Building Trust and Addressing Misinformation

Trust in the healthcare system, media, and the government are some of the most important predictors of vaccine acceptance level in a population. In a June 2020 study, Lazarus et al. found that trust in information from government sources was a critical factor for determining vaccine acceptance levels [10]. Trust in authorities is much worse in the LGBTQ+ communities, potentially due to authorities’ long history of marginalization and systemic discrimination and violence against these communities.

Mass media’s reach, convenience, and speed of dispersion of information have been weaponized for spreading misinformation, myths, and conspiracy theories [80,81,82,83,84]. This has made accessing truthful information and fact checking a challenging task for the general public [84]. The spread of misinformation has been further aggravated by appealing to people’s emotions, evoking emotional responses such as fear, anger, and hatred [85]. For example, in a study by Schuller et al. that simulated a patient’s search on the Google search engine for advice on the potential link between MMR and autism, only 51% of the websites provided correct information [86]. Another study showed that surfing anti-vaccination websites on the internet, even for 5–10 min, negatively impacted vaccination risk perceptions and vaccine acceptance [87].

Media platforms, internet search engines, and social media platforms should provide trustworthy and transparent information. However, these media platforms cannot be solely relied upon to curb biased or incorrect information. Government, healthcare policymakers, and healthcare providers need to create, identify, promote, and inform people about resources to gather fact-checked, verified content, such as the official websites from the CDC, FDA, WHO, UNICEF, The National Network for Immunization Information (NNii), Advisory Committee on Immunization Practices (ACIP), and medical libraries [88]. Health officials can also use these media platforms to deliver specific health information to the targeted audience. For example, providing information on why moderately to severely immunocompromised people with HIV/AIDS should receive a third vaccine dose [89] and directly addressing misinformation such as vaccine’s cost and side effects. In addition, various LGBTQ+ trusted sources such as local supportive LGBTQ+ chapters, advocacy groups, community leaders, and specific public figures could be leveraged as trusted sources by relaying accurate COVID-19 vaccine information.

### 7.3. Behavior Modifiers

Some studies have suggested that behavior modifiers can positively influence vaccine acceptance. For instance, promoting vaccination as a social norm can motivate the public to get vaccinated. This is a similar strategy to that used to encourage voting; after vaccination, people may get a pin, ribbon, or a badge showing support for immunization and acknowledging their vaccination status [90]. A hospital-based study noted an increase in the influenza vaccination rate after healthcare workers started wearing a badge reading “I am vaccinated against influenza to protect you” [91,92]. Social strategizing, including strategic motivations to protect others from infection (altruism), has been shown to improve vaccination behavior [18]. Social networking means that individuals are more likely to get vaccinated if their friends, family members, or members of their social network support vaccination [91]. Decision aids (with a description of treatment options’ benefits and harms, often including numerical estimates of their likelihood or magnitude) can help people to clarify their values on treatment option benefits and harms; and to encourage them to make an informed choice [93].

### 7.4. Patient-Centered Care

It is the responsibility of all the health staff members to make a patient feel safe and respected. To do that, health staff need to be educated about LGBTQ+ specific medical concerns and care. It can be supplemented by training and retraining using LGBTQ+-centric competency modules and online/print guides on communicating with LGBTQ+ patients [94]. Health staff should exercise an empathetic and nonjudgmental approach to build trust, to assure confidentiality, and to provide a safe nondiscriminatory environment for the patient. Healthcare providers should also identify high-risk individuals with concerns such as addiction and signs of abuse and provide the necessary support and tools. Healthcare administrators, providers, and patient advocates should also work together to ensure equitable, safe, nondiscriminatory, and optimal health care for LGBTQ+ patients [95].

### 7.5. Data Collection

The LGBTQ+ population needs to be adequately represented in healthcare research studies to achieve health equity. We need to prioritize sexual orientation and gender identity data collection in COVID-19 vaccine studies and tracking tools. Collection of LGBTQ+ relevant health information will help healthcare providers to understand the LGBTQ+ specific concerns, provide healthcare services, and formulate policies tailored to individual LGBTQ+ needs, including addressing healthcare disparities. Various public health surveillance systems, such as the U.S. Department of Health and Human Services’ and the WHO/United Nations Children’s Fund (UNICEF) Joint Reporting Process (JRF), are used for monitoring COVID-19 immunization data [96]. Data from these tools can help to identify trends and gaps, including vaccine hesitancy and help to formulate appropriate intervention. The federal government and advocacy groups should advocate for the inclusion of sexual and gender identity data, thereby, leveraging pre-existing robust data collection tools to identify and address LGBTQ+ community-specific concerns, including COVID-19-related (vaccine hesitancy) and also other health disparities [43,97]. In addition, vaccine hesitancy is a dynamic, multifaceted problem, and therefore longitudinal surveillance tools can help to design and adapt response strategies and programs when needed.

The collection of gender and sexuality-based survey questions should be optional and anonymized. Studies have reported collecting individually identifiable data as a significant concern for marginalized populations, including LGBTQ+ and undocumented immigrant populations [98,99,100]. Practical measures to prevent any potential breach of protected health information or patient identifiers should be implemented. Violation of such data can have devastating consequences for an LGBTQ+ individual, including increased risk of physical abuse, violence, homelessness, and employment discrimination [99,100,101,102,103]. Patient safety and comfort must always be prioritized over data collection. In addition, various authors have noted a lack of uniformity in identifying sexual orientation and gender identity [27,28,104,105]. It is crucial to establish universal guidelines for identifying and collecting such information to leverage the data effectively.

### 7.6. Public Health and Vaccine Policies

In addition to exploring individual-based approaches, a structural change is needed to address systemic and structural oppression of LGBTQ+ people, including policies against discrimination, unemployment, mass incarceration, health insurance coverage, medical services, including telehealth not just for COVID-19 but also for chronic conditions disproportionately affecting the LGBTQ+ population.

## 8. Limitations

This study has some limitations. There is a high level of heterogeneity in study designs, target populations, enrollment methods, and outcomes. In addition, most of the data reported on the LGBTQ+ population were collected from the USA and Canada. It may limit the applicability of some of these findings to the general population. Our study only included data from English-language articles, and therefore may have missed relevant information from other language-based sources. Additionally, three databases were searched for the evidence, and therefore other sources might have been overlooked. The systematic review protocol was not registered in repositories. Lastly, the systematic review was conducted using cross-sectional surveys. These studies only provide a snapshot of the findings at a specific time, and therefore might not be helpful in predicting future vaccine acceptance or hesitancy rates.

## 9. Conclusions

The LGBTQ+ population has suffered from systemic discrimination, oppression, and structural health inequities. These injustices escalated further during the COVID-19 pandemic. Due to their systematic exclusion from COVID-19 vaccine studies and tracking tools, there are insufficient data on health disparities, including COVID-19 vaccine hesitancy. Limited studies have suggested the most common causes for vaccine hesitancy in the LGBTQ+ population include concern about the COVID-19 vaccine’s safety, efficacy, potential side effects, and previous negative experiences with healthcare providers. To address COVID-19 vaccine hesitancy in the LGBTQ+ population, a collaborative effort between federal governments, healthcare policymakers, healthcare providers, and mass media providers is needed to build trust; listen to and address community-specific concerns; providing uniform, consistent, transparent, and accurate information on vaccine safety and efficacy; and make the vaccine more the accessible including providing safe spaces for vaccination and other healthcare needs.

## Figures and Tables

**Table 1:** Summary of all studies reviewed.

Author	Year	Country	Total Population	NB Gender Present	Number of NB Participants	Overall Gender Distribution	Overall Acceptance Rate (%)	Acceptance Rate in NB Participants (%)
Freeman et al. [9]	2020	UK	5114	Yes	20	M = 2574, F = 2515	71.71%	-
Lazarus et al. [10]	2021	China	712	Yes	0	M = 360, F = 351	88.62%	-
Lazarus et al. [10]	2021	Brazil	717	Yes	4	M = 276, F = 436	85.36%	-
Lazarus et al. [10]	2021	Canada	707	Yes	6	M = 307, F = 392	68.74%	-
Lazarus et al. [10]	2021	Ecuador	741	Yes	10	M = 323, F = 407	71.93%	-
Lazarus et al. [10]	2021	France	669	Yes	2	M = 334, F = 333	58.89%	-
Lazarus et al. [10]	2021	Germany	722	Yes	2	M = 298, F = 417	68.42%	-
Lazarus et al. [10]	2021	India	742	Yes	6	M = 243, F = 485	74.53%	-
Lazaus et al. [10]	2021	Italy	736	Yes	1	M = 323, F = 412	70.79%	-
Lazarus et al. [10]	2021	Mexico	699	Yes	2	M = 332, F = 364	76.25%	-
Lazarus et al. [10]	2021	Nigeria	670	Yes	22	M = 275, F = 373	65.22%	-
Lazarus et al. [10]	2021	Poland	666	Yes	0	M = 362, F = 302	56.31%	-
Lazarus et al. [10]	2021	Russia	680	Yes	6	M = 328, F = 346	54.85%	-
Lazarus et al. [10]	2021	Singapore	655	Yes	3	M = 342, F = 310	67.94%	-
Lazarus et al. [10]	2021	South Africa	619	Yes	3	M = 321, F = 294	81.58%	-
Lazarus et al. [10]	2021	South Korea	752	Yes	0	M = 357, F = 392	79.79%	-
Lazarus et al. [10]	2021	Spain	748	Yes	2	M = 345, F = 401	74.33%	-
Lazarus et al. [10]	2021	Sweden	650	Yes	2	M = 322, F = 326	65.23%	-
Lazarus et al. [10]	2021	UK	768	Yes	14	M = 344, F = 408	71.48%	-
Lazarus et al. [10]	2021	USA	773	Yes	9	M = 337, F = 423	75.42%	-
Reiter et al. [11]	2020	USA	2006	Yes	16	M = 868, F = 1122	68.50%	56.25%
Salali et al. [12]	2020	UK	1088	Yes	31	M = 322, F = 735	82.00%	-
Salali et al. [12]	2020	Turkey	3936	Yes	18	M = 1474, F = 2462	67.00%	-
Shekhar et al. [13]	2020	USA	3479	Yes	7	M = 864, F = 2598	36.00%	43.00%
Teixeira da Silva et al. [14]	2021	USA	1350	Yes	1262	Not reported	Not reported; study focused on predictors of vaccine acceptance	Not reported; study focused on predictors of vaccine acceptance
Bendau et al. [15]	2021	Germany	1779	Yes	10	M = 389, F = 1380	64.50%	-
Kuter et al. [16]	2021	USA	12034	Yes	878	M = 5658, F = 1241	63.70%	28.90%
Lin et al. [17]	2021	Taiwan	1047	Yes	171	M-430, F-617	Sexual minority individuals had higher levels of explicit and intrinsic intention to receive COVID-19 vaccination relative to heterosexual individuals.	Not reported; multiple regression models were used

NB, nonbinary; M, male; F, female.

## Data Availability

Not applicable.
